# Production and evaluation of anti-BP26 monoclonal antibodies for the serological detection of animal brucellosis

**DOI:** 10.3389/fvets.2024.1389728

**Published:** 2024-06-18

**Authors:** Xiaohan Guo, Mingjun Sun, Yu Guo, Yao Wu, Xin Yan, Mengda Liu, Jiaqi Li, Xiangxiang Sun, Xiaoxu Fan, Haobo Zhang, Shufang Sun, Jianlong Wang, Dehui Yin

**Affiliations:** ^1^Key Laboratory of Human Genetics and Environmental Medicine, School of Public Health, Xuzhou Medical University, Xuzhou, China; ^2^Laboratory of Zoonoses, China Animal Health and Epidemiology Center, Qingdao, China; ^3^Testing Laboratory, Inner Mongolia Animal Disease Control Center, Hohhot, China; ^4^College of Animal Science and Technology, Shandong Agriculture University, Taian, China

**Keywords:** brucellosis, competitive ELISA, BP26, diagnosis, monoclonal antibodies

## Abstract

*Brucella* BP26 proves to be a highly immunogenic antigen with excellent specificity in brucellosis detection. In China, the authorized use of the Bp26-deleted vaccine M5ΔBP26 for preventing small ruminant brucellosis highlights the importance of developing accurate detection methods targeting BP26, particularly for the diagnosis of differentiation between infected and vaccinated animals (DIVA). Using the traditional mouse hybridoma technique, we successfully obtained 12 monoclonal antibodies (mAbs) targeting BP26. The efficacy of these mAbs in detecting various animal brucellosis cases using the competitive ELISA method was evaluated. Among them, only the E10 mAb exhibited significant efficiency, being inhibited by 100, 97.62, and 100% of brucellosis-positive sera from cattle, small ruminants, and canines, respectively. The E10-based competitive enzyme-linked immunosorbent assay (cELISA) outperformed the BP26-based indirect enzyme-linked immunosorbent assay (iELISA) in accuracy, particularly for cattle and small ruminant brucellosis, with cELISA sensitivity reaching 97.62% compared to 64.29% for iELISA for small ruminants. Although cELISA showed slightly lower specificity than iELISA, it still maintained high accuracy in canine brucellosis detection. The epitope of mAb E10 was identified in the amino acid sequence QPIYVYPDDKNNLKEPTITGY, suggesting its potential as a diagnostic antigen for brucellosis. In conclusion, the E10-based cELISA presents an effective means of detecting animal brucellosis, particularly significant for DIVA diagnosis in China, where the BP26-mutant vaccine is widely used.

## 1 Introduction

The 26-kDa periplasmic protein of *Brucella*, BP26, serves as a robust immunogenic antigen capable of eliciting an antibody response in *Brucella*-infected animals (1–8). Despite a delayed and weaker antibody response compared to *Brucella* O-polysaccharide (OPS) antigen (3), BP26 exhibits higher specificity in brucellosis detection (9), with minimal cross-reactivity with sera infected with other bacterial pathogens. Additionally, BP26 presents an advantage in detecting brucellosis caused by rough *Brucella* strains, which lack OPS antigen on their surface (4, 10).

Furthermore, studies have demonstrated that BP26 gene-deleted vaccines maintain their protective efficacy against wild-type *Brucella* strains, making BP26-deficient vaccines, such as S19 and Rev1, preferable for achieving serological differentiation between infected and vaccinated animals (DIVA) (11–14). In China, the authorization of the BP26-mutant vaccine M5ΔBP26 for preventing small ruminant brucellosis highlights the importance of developing detection methods targeting BP26 in facilitating DIVA strategies with the widespread use of M5ΔBP26 vaccines (15).

We and other researchers have evaluated the efficacy of the BP26-based indirect enzyme-linked immunosorbent assay (iELISA) in serologically detecting various forms of animal brucellosis (7, 16). Fewer studies have explored the competitive enzyme-linked immunosorbent assay (cELISA) using anti-BP26 monoclonal antibodies (mAbs) for brucellosis detection (17, 18). In this study, we used the traditional mouse hybridoma technique to generate a panel of mAbs targeting BP26. Using a collection of brucellosis-positive sera, we assessed the ability of these mAbs to detect different forms of animal brucellosis, identifying one mAb with high efficiency in cELISA for this purpose. We anticipate that our findings will contribute to the development of novel diagnostic methods for brucellosis.

## 2 Materials and methods

### 2.1 Ethics statement

The animal study was reviewed and approved by the Experimental Animal Ethics Committee of Xuzhou Medical University (approval number: 202208W051).

### 2.2 Production of anti-BP26 mAbs

The BP26 protein (reference strain: 16M/ATCC 23456/NCTC 10094) was expressed using a prokaryotic expression system established in our laboratory (16). The immunization of BALB/c mice and hybridoma screening procedures followed methods outlined in a previously published study (18). Anti-BP26 mAbs were generated by intraperitoneal inoculation of mice with hybridoma cells. Mouse ascites containing mAbs were collected and purified using a commercial Protein G column (Sangon Biotech, Shanghai, China). The purity of the mAbs was assessed using SDS-PAGE.

### 2.3 Sera

All sera utilized in this experiment were archived at the Chinese Animal Epidemiology and Health Center (CAHEC) with well-documented backgrounds. Positive sera were sourced from animals from which wild-type *Brucella* had been isolated, while negative sera originated from animals raised in brucellosis-free zones. A total of 245 sera were included in the study, comprising 96 from small ruminants (naturally infected: 42; negative: 54), 96 from dairy cattle (naturally infected: 44; negative: 52), and 53 from canines (naturally infected: 7; negative: 46). Additionally, 231 sera from ruminants immunized with the M5ΔBP26 vaccine were collected. These sera were used to assess the efficacy of both the iELISA and cELISA methods.

Furthermore, rabbit sera (purchased from Tianjin Biochip Corporation, Tianjin, China) artificially infected with *Y. enterocolitica O9*, *E. coli* (O157:H7, O116), *Salmonella urban*, *Ochrobactrum anthropi*, and *Vibrio cholerae* were utilized to assess the specificity of both the iELISA and cELISA methods. Rabbit sera underwent the same testing procedures similar to those for small ruminants, cattle, and canines, including dilutions. HRP-conjugated goat anti-rabbit IgG (diluted at 1:10,000, Bioworld, MN, United States) was used as the secondary antibody.

### 2.4 The characterization of BP26 epitopes recognized by mAbs

Six linear polypeptides of BP26 were synthesized, and iELISA was conducted as previously described (19). In brief, 100 μL of peptide-KLH conjugate (10 μg/mL) was coated onto microplates (Corning, NY, United States) in 0.1 M of carbonate buffer (pH 9.6) and incubated overnight at 4°C. The plates were then washed three times with phosphate-buffered saline containing 0.05% Tween-20 (PBST) and blocked with 5% skimmed milk for 2 h at 37°C. After washing three times with PBST, 100 μL of purified mAb at 1 μg/mL was added to each well and incubated at 37°C for 1 h with the same volume of normal mouse serum used as a negative control. The plates were washed again three times and then incubated with 100 μL of horseradish peroxidase (HRP)-conjugated recombinant protein G (diluted at 1:10,000) (Bersee, Beijing, China) for 45 min at 37°C. TMB was used as the colorimetric substrate, and the optical density was measured at 450 nm (OD450) using an ELISA plate reader (BioTek, United States). A ratio of OD450 value of mAb to normal mouse sera of above 1.5 was considered a positive result.

### 2.5 iELISA and cELISA

The iELISA was conducted following previously established procedures with slight modifications (16). One change was the concentration of BP26 protein used to coat the microplates, which was adjusted to 1 μg/mL. Additionally, commercial blocking buffer (BioFX, United States) was substituted for 5% skimmed milk.

For cELISA, critical conditions, such as the coating concentration of BP26 and the volume of serum and mAbs, were optimized using checkerboard titration. HRP-conjugated rabbit anti-mouse IgG (diluted at 1:10,000) (Sangon, China) served as the secondary antibody to detect the mAb. The remaining steps were identical to those of the iELISA. All samples were repeated three times.

### 2.6 Statistical analysis

The overall sensitivity and specificity of iELISA and cELISA in detecting animal brucellosis were calculated using receiver operating characteristic (ROC) curves. The optimal cutoff values were defined using the highest sum of sensitivity and specificity. For each optimal cutoff value, the main parameters, such as sensitivity, specificity, positive predictive value (PPV), and negative predictive value (NPV), were calculated. The dot plot was performed using GraphPad Prism version 6.05.

## 3 Results

### 3.1 The production of anti-BP26 mAbs and epitope recognition analysis

After screening, a total of 12 hybridoma cell lines were found to secrete mAbs, which were reactive to BP26. Then, mAbs were prepared from mouse ascites and coded as E1–E12. Based on the results of BP26 polypeptides iELISA, the mAb E10 reacted to the polypeptide of QPIYVYPDDKNNLKEPTITGY and three mAbs coded as E4, E5, and E8 reacted to polypeptide AAAPDNSVPIAAGENSYNVSVNVVFE, while the remaining eight mAbs did not react to any of these polypeptides (Table 1).

**Table 1:** BP26 polypeptides and recognition by mAbs.

No. polypeptides	Sequence of amino acids	Recognized by
P1	AFAQENQMTTQPARIAV	—
P2	KAGIEDRDLQTGGIN	—
P3	QPIYVYPDDKNNLKEPTITGY	E10
P4	GVNQGGDLNLVNDNPSAVIN	—
P5	LSRPPMPMP	—
P6	AAAPDNSVPIAAGENSYNVSVNVVFE	E4, E5, E8

### 3.2 The inhibition of animal brucellosis-positive sera to mAbs

cELISA was conducted individually for cattle, small ruminants, and canine brucellosis-positive sera using these mAbs. The inhibition percentage (IP) for each mAb was calculated as [100 − (OD450 value of positive serum/OD450 value of negative serum) × 100] %. Based on the IP values, the positive sera were categorized into five groups: <20%, 20–39%, 40–59%, 60–79%, and ≥80%.

If a serum with an IP value of more than 20% was positive, E10 inhibited 100% of cattle brucellosis sera, 97.62% of small ruminant sera, and 100% of canine sera (Table 2). With the same brucellosis-positive sera, the other mAbs exhibited significantly lower IPs compared to E10. Similarly, when the threshold value of IP was raised to 40%, E10 still displayed the highest IPs for cattle, small ruminants, and canine brucellosis-positive sera, with values of 38.64, 57.14, and 100%, respectively. Based on these results, E10 was selected as the preferred choice for assembling a BP26-based cELISA kit.

**Table 2:** Number of sera detected by anti-BP26 mAbs under different IP ranges.

Number of sera	Anti-BP26 mAb	Number of sera with different IP ranges					Percentage of sera with IP more than 20% (%)	Percentage of sera with IP more than 40% (%)
		<20%	20–39%	40–59%	60–79%	≥80%		
Cattle (44)	E1	43	1	0	0	0	2.27	0.00
	E2	43	1	0	0	0	2.27	0.00
	E3	42	2	0	0	0	4.55	0.00
	E4	40	2	2	0	0	9.09	4.55
	E5	43	0	1	0	0	2.27	2.27
	E6	34	6	3	1	0	22.73	9.09
	E7	40	2	2	0	0	9.09	4.55
	E8	42	1	1	0	0	4.55	2.27
	E9	42	2	0	0	0	4.55	0.00
	E10	0	27	5	8	4	100.00	38.64
	E11	43	1	0	0	0	2.27	0.00
	E12	36	2	3	3	0	18.18	13.64
Small ruminants (42)	E1	41	1	0	0	0	2.38	0.00
	E2	42	0	0	0	0	0.00	0.00
	E3	41	1	0	0	0	2.38	0.00
	E4	39	1	2	0	0	7.14	4.76
	E5	41	0	1	0	0	2.38	2.38
	E6	28	10	3	1	0	33.33	9.52
	E7	41	1	0	0	0	2.38	0.00
	E8	38	2	2	0	0	9.52	4.76
	E9	42	0	0	0	0	0.00	0.00
	E10	1	17	10	10	4	97.62	57.14
	E11	40	1	1	0	0	4.76	2.38
	E12	32	3	4	3	0	23.81	16.67
Canine (7)	E1	6	0	1	0	0	14.29	14.29
	E2	5	1	1	0	0	28.57	14.29
	E3	6	1	0	0	0	14.29	0.00
	E4	6	1	0	0	0	14.29	0.00
	E5	7	0	0	0	0	0.00	0.00
	E6	3	3	1	0	0	57.14	14.29
	E7	7	0	0	0	0	0.00	0.00
	E8	6	1	0	0	0	14.29	0.00
	E9	7	0	0	0	0	0.00	0.00
	E10	0	0	1	4	2	100.00	100.00
	E11	6	1	0	0	0	14.29	0.00
	E12	3	2	2	0	0	57.14	28.57

### 3.3 The efficiency of the E10-based cELISA in detecting animal brucellosis

To evaluate the efficacy of mAb E10 in detecting animal brucellosis, cELISA was conducted using positive and negative sera from various domestic animals, including cattle, small ruminants, and canines. The optimal coating concentration of BP26 protein was set at 10 μg/mL, and the optimal serum volume used was 30 μL, which was mixed with 70 μL of pre-diluted mAb (0.875 μg/mL) before transferring to each well of the microplate.

**Figure 1:**
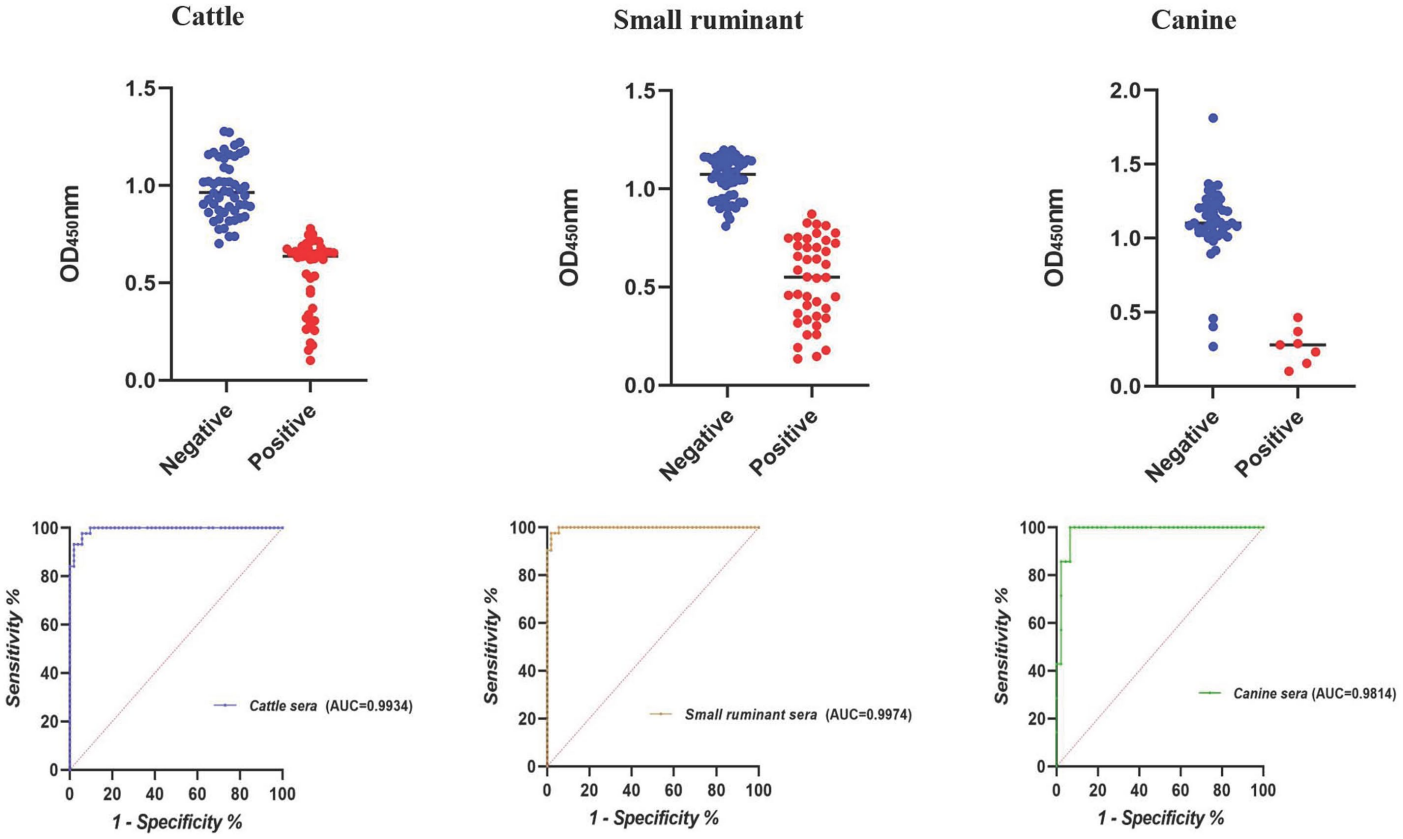
Dot plot and ROC of the E10-based cELISA in detecting animal brucellosis sera.

The largest area under the ROC curve was observed for small ruminant sera (AUC = 0.9974), followed by cattle (AUC = 0.9934) and canine sera (AUC = 0.9814) (Figure 1). Using the optimal cutoff values, the cELISA accuracy for small ruminants was the highest (97.94%), with PPV and NPV recorded as 97.62 and 98.18%, respectively (Table 3). Cattle sera showed similar accuracy, although the PPV was slightly lower than that of small ruminant sera. Canine sera exhibited the highest PPV (100%), but due to the limited number of samples, the NPV was the lowest, resulting in the lowest overall accuracy among the three groups of sera.

**Table 3:** PPV and NPV of the E10-based cELISA in detecting animal brucellosis sera.

Cutoff value	Positive		Negative		PPV (%)	NPV (%)	Accuracy (%)
	TP	FN	TN	FP			
<0.7283 (cattle)	41	3	51	1	93.18	98.07	95.83
<0.8370 (small ruminant)	41	1	54	1	97.62	98.18	97.94
<0.6973 (canine)	7	0	43	3	100	93.48	94.34

TP, true positives; TN, true negatives; FP, false positives; FN, false negatives; accuracy, (TP + TN)/(TP + FN + TN + FP) × 100; PPV, positive predictive value TP/(TP + FP) × 100; NPV, negative predictive value TN/(TN + FN) × 100.

### 3.4 The efficiency of the BP26-based iELISA in detecting animal brucellosis

Compared to cELISA, the same collections of brucellosis-positive and brucellosis-negative sera were also tested by the BP26-based iELISA. The optimal coating concentration of BP26 protein for iELISA was 0.3 μg/mL, much lower than cELISA.

**Figure 2:**
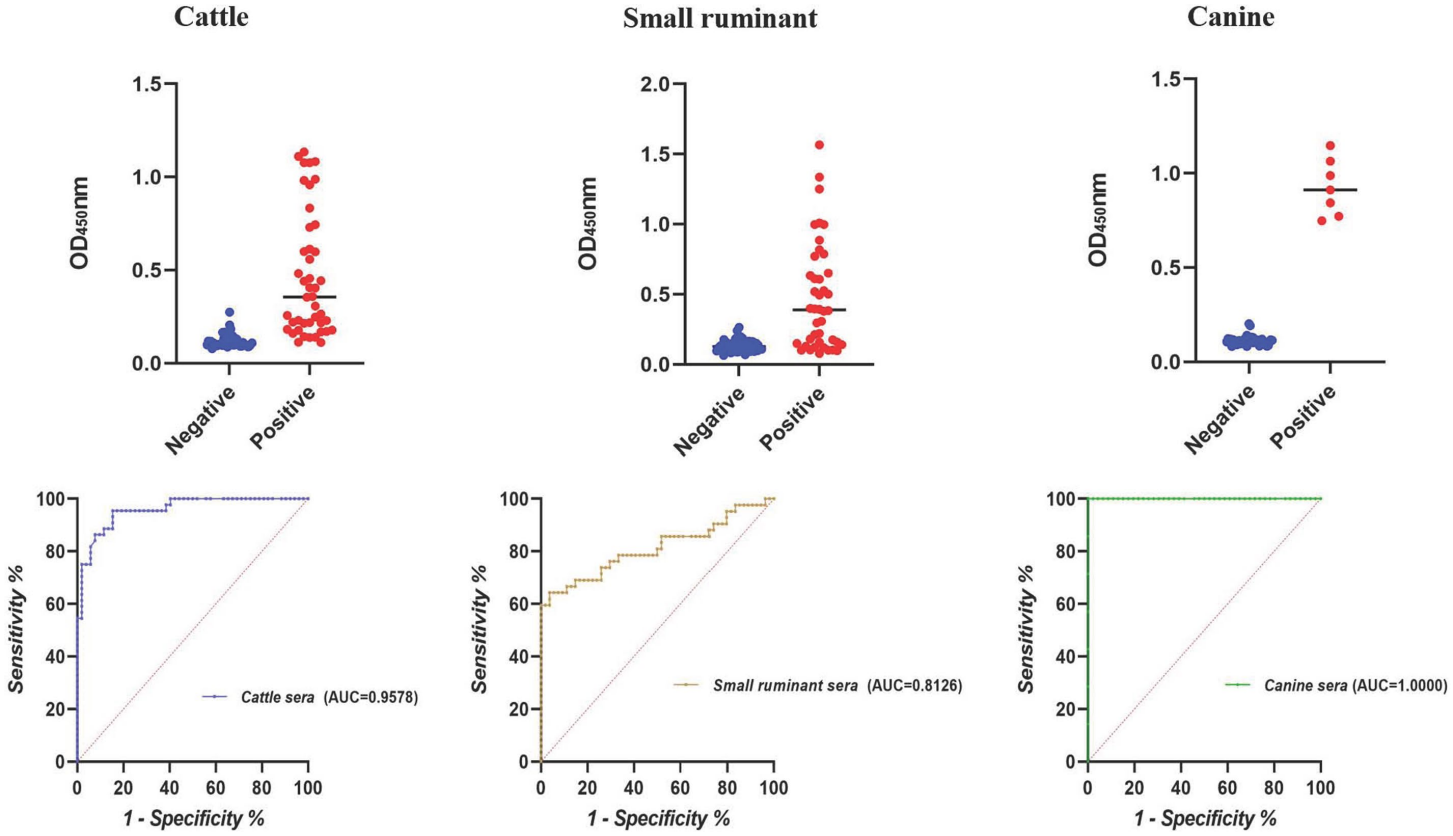
Dot plot and ROC of the BP26-based iELISA in detecting animal brucellosis sera.

According to the result of iELISA, the largest area under the ROC curve was obtained for canine sera (AUC = 1.0000), followed by cattle (AUC = 0.9578) and small ruminant sera (AUC = 0.8126) (Figure 2). Using the optimal cutoff values, the accuracy of cELISA for canine sera was the highest (100%), meaning that all the positive and negative sera were correctly distinguished. Lower accuracy values were obtained for cattle and small ruminant sera (89.58 and 82.30%, respectively) (Table 4).

**Table 4:** PPV and NPV of the BP26-based iELISA in detecting animal brucellosis sera.

Cutoff value	Positive		Negative		PPV (%)	NPV (%)	Accuracy (%)
	TP	FN	TN	FP			
<0.1362 (cattle)	42	2	44	8	95.45	84.61	89.58
<0.2040 (small ruminant)	27	15	52	2	64.29	96.30	82.30
<0.4753 (canine)	7	0	46	0	100.00	100.00	100.00

### 3.5 Cross-reaction to other serum

If a ratio of S/N (OD_450_, sample/negative) >2.0 in iELISA or N/S >2.0 in cELISA was considered to be positive, neither E10-based cELISA nor BP26-based iELISA identified the rabbit sera-infected by *Y. enterocolitica O9*, *E. coli* (*O157:H7*, *O116*), *Salmonella urban*, *Ochrobactrum anthropi,* and *Vibrio cholerae*.

## 4 Discussion

Among the protein antigens utilized for brucellosis detection, BP26 has been extensively studied. Previous research has shown that the BP26-based iELISA can effectively detect various cases of animal brucellosis. However, our experiment suggests that cELISA using anti-BP26 mAbs may be more efficient. Overall, the cELISA method established in this study outperformed iELISA, particularly in terms of accuracy for detecting cattle and small ruminant sera (Tables 3, 4). Notably, there was a significant difference in small ruminant detection between cELISA (97.62%) and iELISA (64.29%). Conversely, iELISA showed higher accuracy in detecting canine brucellosis, achieving 100% accuracy as compared to 94.34% for cELISA. This difference primarily stemmed from slightly lower specificity in cELISA, although sensitivity remained comparable to iELISA. Unfortunately, the limited availability of brucellosis-positive sera in canine in our experiment reflects the insufficient attention given to canine brucellosis in China, resulting in a shortage of qualified serum samples. Thus, further evaluation is necessary to assess the efficacy of anti-BP26 mAb-based cELISA in detecting rough *Brucella*-induced brucellosis.

BP26 is an immunogenic protein capable of stimulating various mAbs targeting different epitopes within the mouse model (17, 18). In our experiment, mAb E10 demonstrated significant potential in detecting animal brucellosis, while mAbs E6 and E12 showed some ability but did not meet the necessary sensitivity and specificity requirements for diagnostics. The remaining nine mAbs were largely ineffective as diagnostic reagents. As mAb E10 reacted to the polypeptide QPIYVYPDDKNNLKEPTITGY, its recognized epitope likely lies within this sequence. Interestingly, Qiu et al. (18) reported two epitopes recognized by anti-BP26 mAbs, one of which, with the sequence QPIYVYPD, overlaps with the polypeptide used in our experiment. Although we cannot confirm their exact identity, the antigenicity of the QPIYVYPDDKNNLKEPTITGY sequence suggests that it may be a useful diagnostic antigen for brucellosis. Furthermore, this proposed epitope falls within the BP26 region as described by Seco-Mediavilla et al. (20), between amino acids 55 and 152, which showed superior specificity in detecting small ruminant sera. This finding aligns with our data, indicating that the E10-based cELISA also exhibits high specificity for brucellosis in small ruminants.

Currently, widespread vaccination with attenuated *Brucella* live vaccines is a key strategy for controlling animal brucellosis in high-prevalence areas, with vaccines possessing DIVA competency being particularly favored by authorities. In China, the commercialization and increasing utilization of the M5ΔBP26 vaccine, which contains a Bp26 deletion, for immunizing small ruminants are notable. The corresponding iELISA utilizing BP26 as an antigen has been proposed to differentiate between M5ΔBP26 vaccinated sera and naturally infected ones (21). Given that the mAb E10-based cELISA established in this study demonstrated better sensitivity and specificity than iELISA, our unpublished data showed that all of the small ruminant sera collected from 7 days to 207 days after vaccination by M5ΔBP26 were not recognized by this cELISA method, demonstrating that the cELISA might be more suitable for the diagnosis of DIVA.

In conclusion, brucellosis remains a significant zoonotic disease, particularly in China, where it is highly endemic in certain regions. The cELISA and iELISA methods outlined in this study not only facilitate the detection of brucellosis in cattle, small ruminants, and canines but also serve as DIVA tests when the Bp26-deleted vaccine is administered. It is hoped that the vaccination and DIVA test strategy will expedite the eradication of animal brucellosis in China.

### 4.1 Limitations

Although our constructed cELISA effectively distinguishes animal brucellosis sera from non-brucellosis sera and can differentiate between immune sera and naturally infected sera in ruminants, our study has certain limitations. First, the sample size is limited, and expanding it will require verifying the sensitivity and specificity of the constructed method. Second, the M5ΔBP26 vaccine strain is specific to China, limiting the applicability of our method to distinguish vaccine-immune sera, which is used worldwide. Finally, our study only included small ruminants immunized sera, and further investigation is needed to assess its ability to differentiate between immunized and naturally infected sera in other animals.

## Data availability statement

The original contributions presented in the study are included in the article/Supplementary material, further inquiries can be directed to the corresponding authors.

## Ethics statement

The animal study was approved by Experimental Animal Ethics Committee of Xuzhou Medical University. The study was conducted in accordance with the local legislation and institutional requirements.

## Author contributions

XG: Conceptualization, Data curation, Writing – original draft. MS: Conceptualization, Writing – original draft. YG: Conceptualization, Writing – original draft. YW: Writing – review & editing. XY: Writing – review & editing. ML: Writing – review & editing. JL: Writing – review & editing. XS: Writing – review & editing. XF: Writing – review & editing. HZ: Funding acquisition, Writing – review & editing. SS: Conceptualization, Writing – review & editing. JW: Conceptualization, Writing – review & editing. DY: Funding acquisition, Methodology, Writing – review & editing.

## Supplementary material

Supplementary TABLE 1 Raw data for Figure 1.

Supplementary TABLE 2 Raw data for Figure 2.

Supplementary TABLE 3 Details of the production of BP26 antigen (epitope) and monoclonal antibodies.
