# Supplementary material for: Production and evaluation of anti-BP26 monoclonal antibodies for the serological detection of animal brucellosis
Source: Front Vet Sci. 2024 Jun 18;11:1389728. doi: 10.3389/fvets.2024.1389728 (PMC11217538; doi:10.3389/fvets.2024.1389728)
Supplement: Supplementary TABLE 3 — Details of the production of BP26 antigen (epitope) and monoclonal antibodies. [file Table_3.docx]

Details of the production of BP26 antigen (epitope) and monoclonal antibodies

1. Production of BP26 antigen (epitope)

BP26 antigen (epitope) was prepared in-house by the group and its details are given below[1, 2]:

1.1 Prediction and Synthesis of PeptideEpitopes of *Brucella melitensis* BP26

NCBI website to obtain the BP26 gene sequence of *B. melitensis* 16S strain, use MEGA to analyze the homologous recombination sequence and prokaryotic expression. The conserved amino acid sequences of these proteins were used to predict B cell epitopes using BepiPred tool in IEDB (http://tools.iedb.org/bcell/). Prediction threshold is 0.350(default value), above this threshold is possible epitope. Peptides longer than 6 amino acids were assumed as effective epitope and selected. Each of selected B cell epitope was chemically synthesized and coupled to keyhole limpet hemocyanin (KLH) in Sangon Biotech Company (Shanghai, China). The purity of each polypeptide-KLH was more than 90%.

1.2 Construct BP26 prokaryotic expression gene sequence and construct prokaryotic expression system.

1.2.1 Synthesize target gene

The target gene was synthesized by BP26, and pET30a expression plasmid was constructed. The constructed expression plasmid was transferred into expression vector BL21 for IPTG-induced expression.

1.2.2 Bulk expression

(1) Add 50 μl of activated bacterial solution into 5 mL of corresponding resistant LB liquid medium, incubate at 37℃, 200 rpm.

(2) Transfer the cultured bacterial solution to 200 mL of the corresponding resistant LB liquid medium, 37℃, 200 rpm, culture until OD=0.6-0.8, IPTG (0.5 mM) 16℃ induction overnight.

(3) Bacterial collection: centrifuge at 6000 rpm for 8 min, discard the supernatant.

(4) Ultrasonic breaking: the bacteria were blown apart with 20-30 mL of 10 mM Tris-HCl (pH 8.0) solution, and ultrasonically broken (500 W , 180 times, 5 s each time, 5 s interval).

1.2.3 Protein purification (washing of inclusion bodies)

(1) 20~30 mL 10 mM Tris-HCl (pH8.0) solution resuspend the precipitate obtained by ultrasonic centrifugation, and leave it for 10 min.

(2) 12000 rpm, centrifuge for 10 min, and transfer the supernatant to another tube for storage.

(3) Resuspend the precipitate in 20~30 mL of 10 mM Tris-HCl (pH 8.0) solution and leave for 10 min.

(4) Centrifuge at 12000 rpm for 10 min and discard the supernatant.

(5) Repeat (3) and (4) once.

(6) Add a small amount of 10 mM Tris-HCl (pH 8.0) solution to resuspend the precipitate, then add 5~10 mL of 10 mM Tris-HCl (pH 8.0) solution containing 8 M urea to dissolve the protein.

(7) 12000 rpm, centrifuge for 10 min, collect the supernatant and take 50 μl for electrophoresis.

1.2.4. Protein purification (denaturing nickel column)

(1) Wash the nickel column (Ni Sepharose 6 Fast Flow, GE Healthcare) with deionized water to pH 7.0.

(2) Hang nickel to pH 2~3.

(3) Wash the column with deionized water to pH 7.0.

(4) Equilibrate the nickel column with 10 mM Tris-HCl (pH 8.0) solution, about 100 mL.

(5) Equilibrate the nickel column with 10 mM Tris-HCl (pH 8.0) solution containing 8 M urea and 0.5 M NaCl, about 50 mL.

(6) Dilute the sample on the sample. The sample contained 0.5 M NaCl, 8 M urea, and 10 mM Tris-HCl (pH 8.0).

(7) At the end of the sample uptake, the column was washed with 10 mM Tris-HCl (pH 8.0) solution containing 8 M urea, 0.5 M NaCl.

(8) The column was eluted with 10 mM Tris-HCl (pH 8.0) solution containing 15 mM imidazole, 60 mM imidazole, and 500 mM imidazole (containing 8 M urea, 0.5 M NaCl), and the protein peaks were collected separately.

(9) Protein purification was detected by electrophoresis, and protein concentration was detected by BCA protein quantification kit.

2. BP26 monoclonal antibody preparation

BP26 monoclonal antibody reference was prepared as detailed below[3]:

2.1 Mouse immunization

(1) Four BALB/c female mice were numbered 1, 2, 3 and 4. Before each immunization, 2 μL of blood was collected from the tail vein of each mouse, added to an EP tube with 198 μL of PBS added beforehand, mixed thoroughly, and labeled with the date, etc., and stored at -20 ℃ for subsequent ELISA testing of the immunization serum potency;

(2) Take a 1 mL syringe and perform multi-spot immunization on the subcutaneous dorsum, groin, and foot pads, respectively, with a volume of 30ug of protein/each immunization. After the immunization interval of 5-10 days, blood was taken from the tail vein, and the results of serum potency were determined by ELISA, the last two immunizations could be done by intraperitoneal injection with bacterial solution (especially the last immunization, which should be done 4 days before cell fusion);

2.2 i-ELISA Detection of Immune Potency:

(1) BP26 antigen was coated onto ELISA plates at ratios of 200:1, 400:1, 800:1, and further diluted to 102400:1, with 100 μL per well, and incubated overnight at 4°C.

(2) Discard the coating solution, tap the plate, and wash with 300 μL/well of PBST (PBS with 1 mL Tween-20% added to 1000 mL PBS) for 3 times, 3 minutes each, with shaking.

(3) Add 100 μL of PBS to each well. In the first row, add 100 μL of serum collected from immunized mice, with each serum applied to 2 wells at a time. Mix the first row using a multichannel pipette, transfer 100 μL to the second row, and repeat this process for serial dilution, doubling each time. Finally, discard 100 μL from the last row, and incubate at 37°C for 1 hour. Negative control can be PBS, while positive control can be 2 μL of positive blood added to a well containing 200 μL of PBS.

(4) Discard the liquid, tap the plate, wash with 300 μL/well of PBST for 3 times, 3 minutes each, and add 100 μL of secondary antibody solution per well (secondary antibody: PBS = 1:15000), incubate at 37°C for 1 hour.

(5) Discard the liquid, tap the plate, wash with 300 μL/well of PBST for 3 times, 3 minutes each, and add 100 μL of TMB color developing solution per well, reacting at room temperature in the dark for 15 minutes.

(6) Add 50 μL of stop solution to each well, and measure the OD450nm using a microplate reader.

2.3 Monoclonal Antibody Preparation Technique

2.3.1 Preparation of Myeloma Cells SP2/0

Thaw the frozen myeloma cell line SP2/0 rapidly within 1 minute, centrifuge at 1500 rpm for 5 minutes, discard the liquid, resuspend in 1640 culture medium, transfer to cell culture bottles pre-filled with 20% serum-containing 1640 medium, and observe cell adhesion overnight in a constant temperature incubator at 37°C with 5% CO2.

2.3.2 Preparation of Feeder Layer Cells

(1) Obtain blood from the eyeball of a healthy, non-immunized BABL/c mouse, store at -20°C for use as a negative control.

(2) Euthanize the mouse at the cervical vertebra, disinfect the entire body by soaking in 75% alcohol for 5 minutes.

(3) Illuminate the ultraclean bench with UV light for 30 minutes, prepare 1640 medium containing 2% HAT and 20% serum.

(4) Place the disinfected mouse in a glass dish on the ultraclean bench. Using scissors, gently make a small incision in the abdominal skin of the mouse (be careful not to rupture the peritoneum). Tear open the abdominal skin with both hands to completely expose the abdominal cavity.

(5) Using a 5 mL syringe, inject serum-free 1640 medium into the mouse's abdominal cavity along the bottom fat layer, inject 10 mL.

(6) Gently massage the mouse's abdomen with the syringe plunger to thoroughly mix macrophages in the 1640 medium.

(7) Aspirate the injected 1640 medium gently along the injection site into a centrifuge tube. Centrifuge at 1500 rpm for 5 minutes. Resuspend in 2% HAT and 20% serum-containing 1640 medium and count the cells.

(8) Adjust the cell concentration to 10^5 cells/mL, calculate the volume of 2% HAT and 20% serum-containing 1640 medium to add.

(9) Seed a 96-well plate with 100 μL per well, and observe cell adhesion overnight in a constant temperature incubator at 37°C with 5% CO_2_.

2.3.3 Cell Fusion

(1) Preparation of Splenocyte Suspension from Immunized Mice

Four days before cell fusion, immunize BALB/c mice with high serum antibody titers once (bacterial suspension), collect blood from the eyeball, isolate positive serum, euthanize at the cervical vertebra, and disinfect by soaking in 75% alcohol for 5 minutes. Aseptically remove the spleen on the ultraclean bench. Rinse the spleen in a petri dish containing serum-free RPMI-1640 medium. Grind the tissue in a culture dish, washing with RPMI-1640 medium while grinding, filter the tissue suspension through a sieve, collect the cell suspension in a 50 mL centrifuge tube, centrifuge at 1500 rpm for 10 minutes, discard the supernatant, and resuspend in serum-free medium. Adjust the cell concentration to 10^7 cells/mL using a hemocytometer and set aside.

(2) Preparation of Myeloma Cells

Take SP2/0 cells in the logarithmic growth phase, centrifuge at 1500 rpm for 10 minutes, discard the supernatant, appropriately dilute with 1640 medium, count the cell concentration, and adjust the cell concentration to 10^6 cells/mL for later use. (3) Cell Fusion

1) Take an appropriate number of splenocytes and SP2/0 cells according to the quantity prepared for feeder layer cells (splenocytes: SP2/0 = 10:1).

2) Thoroughly mix the two types of cells, centrifuge and discard the supernatant. This step can be repeated twice to ensure thorough mixing of the cells.

3) After discarding the liquid, use fingers to gently flick the cells at the bottom of the tube to create a homogenate.

4) Slowly add 1 mL of 50% preheated PEG at 37°C while gently shaking. Add within 1 minute.

5) Let it stand for 90 seconds, slowly add preheated incomplete culture medium to terminate the PEG reaction. Add at a rate of 1 mL in the 1st minute, 2 mL in the 2nd minute, 3 mL in the 3rd minute. When adding around 20 mL slowly, gently mix and centrifuge at 1500 rpm for 5 minutes and discard the supernatant.

6) Gently mix in 2% HAT culture medium, disperse into single-cell suspension, ensuring gentle handling to avoid dispersing the fused cells. Add 2% HAT culture medium to the required volume and mix well.

7) Add 100 μL per well to the pre-prepared 96-well culture plate containing feeder cells. Label and culture in a constant temperature incubator at 37°C with 5% CO2 for 5 days without disturbance.

(4) Selection of Hybridoma Positive Cell Lines

Observe the growth status of cells daily after 5 days of cell fusion. For wells with presence of fused cells (cells covering 1/3~1/4 of the bottom of the well), use i-ELISA method for positive hybridoma cell line selection (usually screened 2-3 times to ensure accuracy). At the same time, semi-quantitatively change to 2% HT culture medium to accelerate the growth of cell colonies. On the 13th day, change half of the medium once.

(5) Cloning of Hybridoma Cells

Subclone the cells from positive wells using a limiting dilution method. The specific steps are as follows:

1) Prepare feeder cells one day before cloning hybridoma cells.

2) After screening positive culture wells by i-ELISA, aspirate the cells with 20% serum-containing 1640 medium, mix well, transfer all cell suspension to a sterile small flask, and count the cells using a hemocytometer. Take some cells for cloning culture and transfer the remaining cells to a 24-well plate for expansion culture.

3) Dilute the positive clone cell suspension to 1 cell/100 µL using 20% newborn calf serum-containing 1640 medium, mix well, and add dropwise to a 96-well cell culture plate. Incubate at 37°C with 5% CO2. Each well should contain only one cell, facilitating monoclonal formation. During the cloning process, attention should be paid to the expansion culture and cryopreservation of positive hybridoma cell lines.

(6) Expansion Culture of Hybridoma Cells

When the positivity rate of hybridoma cells reaches 100%, select wells with high OD450 values for focused culture. When the cells cover the bottom of the well, transfer the cells from the 96-well cell plate to a 24-well cell plate for passage culture. Passage the cells until reaching 4-6 wells, then transfer them to cell culture flasks for further culture (i-ELISA can be performed simultaneously to assess the strength of positivity; sometimes, with increasing passages, positive clones may weaken or even disappear).

(7) Cryopreservation of Hybridoma Cells

After each cloning, the remaining positive hybridoma cells should be cryopreserved. Additionally, after expanding the hybridoma cell line secreting monoclonal antibodies, it should also be cryopreserved. Gently aspirate the cells from the culture bottle wall into a centrifuge tube, centrifuge at 1500 rpm for 5 minutes, discard the supernatant, and collect the cell pellet. Add an appropriate amount of cell cryopreservation solution, gently mix the cell pellet, and aliquot into cryovials. Place the cryovials in a refrigerator at 4°C for 0.5 hours, then in a -20°C freezer for 1.5 hours, and finally store in a -80°C ultra-low temperature freezer (if not in use for a long time, transfer to liquid nitrogen after overnight storage in a -80°C ultra-low temperature freezer).

2.3.4 Preparation of Ascites Fluid for Monoclonal Antibodies

Ascites fluid is prepared using in vivo induction method. One week before inoculating positive hybridoma cells, inject each healthy female BALB/c mouse aged 8-12 weeks with 0.5 mL of liquid paraffin oil in the abdominal cavity. After one week, gently aspirate the positive hybridoma cells from the culture bottle wall into a centrifuge tube and centrifuge at 1500 rpm for 10 minutes. Resuspend the cell pellet in PBS solution, adjust the cell density to 1x10^7 cells/mL, and inject 0.5 mL of cell suspension into the abdominal cavity of each mouse, approximately 5x10^6 cells. After inoculation, monitor the condition of the mice. When the abdomen of the mouse significantly enlarges and its movement slows down, start collecting ascites fluid. Use a 5 mL syringe to extract the fluid from the abdominal cavity. The fluid may be light yellow or dark red. Centrifuge at 4°C overnight at 10000 rpm for 10 minutes to remove blood cells and lipid clusters, collect the supernatant as ascites fluid, label and aliquot it, and store at -20°C. Collect ascites fluid every 2 to 3 days when it accumulates again. Each mouse can usually be tapped 2-3 times, referred to as the 1st, 2nd, and 3rd generation ascites fluid.

2.4 BP26 Monoclonal Cell Subtype Identification

Positive cell strains selected were identified using the SouthernBiotech Mouse Antibody Subtype Identification Kit, following the instructions as outlined below:

(1) Dilute subclass-specific antibodies in coating buffer to a final concentration of 2 μg/mL, 100 μL per well, and incubate overnight at 4°C. Wash three times with PBST wash buffer.

(2) Block with 2% skim milk blocking solution, 200 μL per well, incubate at 37°C for 2 hours, then wash three times with PBST wash buffer.

(3) Add primary antibody (cell culture supernatant) and negative control (SP2/0 culture supernatant), 100 μL per well, incubate at 37°C for 1 hour, then wash three times with PBST wash buffer.

(4) Dilute subclass-specific secondary antibodies (Goat Anti-Mouse IgM, IgG1, IgG2a, IgG2b, etc.) in PBS, 100 μL per well, add to appropriate wells, incubate at 37°C for 1 hour, then wash three times with PBST wash buffer.

(5) Add chromogenic substrate, 100 μL per well, and incubate for approximately 10 minutes.

(6) Stop the reaction by adding 50 μL of stop solution to each well.

(7) Measure absorbance at dual wavelengths (450 nm, 630 nm) and record and save the data.

2.5 Purification of Mouse Ascites Fluid

(1) Purify Protein G from ascites fluid using Shanghai Bio-work Protein G Prepacked Gravity Columns. First, equilibrate the columns with 5 column volumes of Binding/Wash Buffer to ensure the resin inside the column is in the same buffer volume as the target protein. Repeat this step 2-3 times.

(2) Add the sample to the pre-equilibrated gravity column and allow it to incubate for at least 2 minutes to ensure thorough contact between the sample and the resin inside the column. Collect the flow-through, and repeat the sample application to increase binding efficiency.

(3) Wash the column with 10-15 column volumes of Binding/Wash Buffer to remove nonspecifically bound proteins, and collect the wash fractions.

(4) Elute using 5-10 column volumes of Elution Buffer, collecting fractions for analysis. Each fraction should be eluted into a separate tube to ensure all bound target proteins are eluted while obtaining high purity and concentration of protein. Eluted fractions should be immediately neutralized with neutralization buffer.

(5) Measure the purified antibody concentration using the BCA method and determine antibody potency and subtype using i-ELISA.

References

1. Yin D, Bai Q, Wu X, Li H, Shao J, Sun M, et al. Correction: Paper-based ELISA diagnosis technology for human brucellosis based on a multiepitope fusion protein. PLoS Negl Trop Dis. 2021;17(1):e0011079.

2. Bai Q, Li H, Wu X, Shao J, Sun M, Yin D. Comparative analysis of the main outer membrane proteins of Brucella in the diagnosis of brucellosis. Biochem Biophys Res Commun. 2021; 560:126-31.

3. Qiu J, Wang W, Wu J, Zhang H, Wang Y, Qiao J, et al. Characterization of periplasmic protein BP26 epitopes of Brucella melitensis reacting with murine monoclonal and sheep antibodies. PLoS One. 2012;7(3):e34246.
